# Reply to "comment on: Vegetable peptones as a fetal bovine serum substitute in human deciduous tooth pulp stem cell culture"

**DOI:** 10.31744/einstein_journal/2026CE2259

**Published:** 2025-12-05

**Authors:** Marizia Trevizani, Laís Lopardi Leal, Silvioney Augusto da Silva, Claudio Gallupo Diniz, Fabiano Freire Costa, Jair Adriano Kopke de Aguiar, Carlos Magno da Costa Maranduba

**Affiliations:** 1 Universidade Federal de Juiz de Fora Institute of Biological Sciences Department of Biology Juiz de Fora MG Brazil Department of Biology, Institute of Biological Sciences, Universidade Federal de Juiz de Fora, Juiz de Fora, MG, Brazil.; 2 Universidade Federal de Juiz de Fora Institute of Biological Sciences Department of Parasitology Juiz de Fora MG Brazil Department of Parasitology, Microbiology and Immunology, Institute of Biological Sciences, Universidade Federal de Juiz de Fora, Juiz de Fora, MG, Brazil.; 3 Universidade Federal de Juiz de Fora Faculdade de Farmácia Department of Pharmaceutical Sciences Juiz de Fora MG Brazil Department of Pharmaceutical Sciences, Faculdade de Farmácia, Universidade Federal de Juiz de Fora, Juiz de Fora, MG, Brazil.; 4 Universidade Federal de Juiz de Fora Institute of Biological Sciences Department of Biochemistry Juiz de Fora MG Brazil Department of Biochemistry, Laboratory of Glycoconjugate Analysis, Institute of Biological Sciences, Universidade Federal de Juiz de Fora, Juiz de Fora, MG, Brazil.

Dear Editor,

We thank the authors for their thoughtful and constructive comments on our study assessing the use of vegetable-derived peptones as substitutes for fetal bovine serum (FBS) in human deciduous dental pulp stem cell cultures. We agree that replacing FBS is essential to address ethical, biosafety, and batch variability concerns that are specifically relevant to translational and clinical applications in cell-based therapies.^( 1 , 2 )^

Consistent with the points raised, our findings demonstrated that wheat peptone supported cell proliferation and osteogenic differentiation at levels comparable to or exceeding those achieved with FBS.^( 3 , 4 )^ This may be attributed to its favorable amino acid composition, specifically its high glutamic acid and proline content. Additionally, plant-derived peptones enhance reproducibility and standardization in culture conditions, facilitating scalability and regulatory alignment for advanced therapeutic applications.^( 2 , 3 )^

Moreover, we acknowledge that further assessments are required to address the following aspects highlighted in this commentary:

1. Long-term functional stability includes the maintenance of stemness and primary biological functions, such as immunomodulatory and neurotrophic capacities.
2. Applicability across different stem cell populations because distinct progenitor cell types may require specific nutritional and signaling cues.
3. Elucidation of molecular mechanisms, specifically how specific peptone compositions affect cellular signaling pathways and the secretome.^( 3 , 4 )^

Ongoing studies in our laboratory are focused on assessing these parameters to strengthen the mechanistic and translational basis for the clinical implementation of plant-based peptone supplementation.

We appreciate the author's contribution to the discussion and reaffirm that incorporating vegetable peptones—specifically wheat peptone—represents a biologically effective, ethically sound, and reproducible alternative to FBS, consistent with current regenerative and translational medicine priorities.^( 1 - 4 )^

## Data Availability

The underlying content is contained within the manuscript.
